# Current treatment decisions in cardiac transthyretin amyloidosis: a multicentre analysis

**DOI:** 10.1007/s00392-026-02848-z

**Published:** 2026-01-26

**Authors:** Daniel Lavall, Katharina Knoll, Sebastian Spethmann, Katrin Hahn, Gina Barzen, Ephraim B. Winzer, Stefanie Jellinghaus, Lisa K. Schöner, Monique Tröbs, Dominik Kauffmann, Nora Donhauser, Lars Michel, Julia Vogel, Tienush Rassaf, Maria Papathanasiou, Lara S. Schlender, David M. Leistner, Birgit Aßmus, Bernhard Unsöld, Larissa Bühner, Fabian aus dem Siepen, Eva Hofmann, Christian Nagel, Ingrid Kindermann, Angela Zimmer, Roman Pfister, Matthieu Schäfer, Natascha Majunke, Irina Müller-Kozarez, Heribert Schunkert, Patrick Fuchs, Stéphanie K. Schwarting, Yuliyan Metodiev, Selen Alieva, Ali Yilmaz, Alexandru Zlibut, Julian Mustroph, Maria Tafelmeier, Thomas Krammer, Stefan Störk, Aikaterini Papagianni, Maximilian J. Steinhardt, Vladimir Cejka, Caroline Morbach, Teresa Trenkwalder

**Affiliations:** 1 Department of Cardiology, Leipzig University Hospital, Leipzig, Germany; 2 Department of Cardiology, German Heart Centre Munich, TUM University Hospital, School of Medicine and Health, Technical University of Munich, Munich, Germany; 3 German Center for Cardiovascular Research (DZHK E.V.), Partner Site Munich Heart Alliance, Munich, Germany; 4 Department of Cardiology, Angiology and Intensive Care Medicine, Deutsches Herzzentrum Der Charité (DHZC), Campus Charité Mitte, Berlin, Germany; 5 Charité – Universitätsmedizin Berlin, corporate member of Freie Universität Berlin and Humboldt Universität Zu Berlin, Campus Charité Mitte, Berlin, Germany; 6 Amyloidosis Center Charité Berlin (ACCB), Amyloidosis Center of Charité, Campus Charité Mitte, Campus Benjamin Franklin and Campus Virchow Klinikum, Berlin, Germany; 7 DZHK (German Centre for Cardiovascular Research), Partner Site Berlin, Germany; 8 Department of Neurology and Experimental Neurology, Charité Universitätsmedizin Berlin corporate member of Freie Universität Berlin and Humboldt Universität Zu Berlin, Campus Benjamin Franklin, Berlin, Germany; 9 Berlin Institute of Health at Charité – Universitätsmedizin Berlin, Berlin, Germany; 10 Department for Internal Medicine and Cardiology, Heart Centre Dresden, Technische Universität Dresden, University Hospital, Dresden, Germany; 11 Department of Medicine 2 - Cardiology and Angiology, Friedrich-Alexander-Universität Erlangen-Nürnberg, Erlangen, Germany; 12 Department of Cardiology and Vascular Medicine, West German Heart and Vascular Center, University Hospital Essen, Essen, Germany; 13 West German Amyloidosis Center, University Hospital Essen, Essen, Germany; 14 Department of Cardiology, University Hospital Frankfurt, Frankfurt, Germany; 15 DZHK, Partner Site Rhine-Main, Frankfurt, Germany; 16 Department of Cardiology and Angiology, UKGM Standort Giessen, Justus-Liebig University Giessen, Giessen, Germany; 17 Department of Cardiology, Angiology and Respiratory Medicine, University Hospital of Heidelberg, Heidelberg, Germany; 18 Department of Internal Medicine III - Cardiology, Angiology and Intensive Care Medicine, University Hospital Saarland, Saarland University, HomburgSaar, Germany; 19 Department III for Internal Medicine, University of Cologne, Faculty of Medicine and University Hospital Cologne, Cologne, Germany; 20 Department of Medicine I, LMU University Hospital, Ludwig Maximillian University of Munich, Munich, Germany; 21 Department of Cardiology I, Division of Cardiovascular Imaging, University Hospital Münster, Münster, Germany; 22 Department of Internal Medicine II, University Medical Centre Regensburg, Regensburg, Germany; 23 Department Clinical Research and Epidemiology, Comprehensive Heart Failure Center, University Hospital Würzburg, Würzburg, Germany; 24 Interdisciplinary Amyloidosis Center of Northern Bavaria, University Hospital Würzburg, Würzburg, Germany; 25 Department Medicine I, University Hospital Würzburg, Würzburg, Germany; 26 Department of Neurology, University Hospital Würzburg, Würzburg, Germany; 27 Department Medicine II, University Hospital Würzburg, Würzburg, Germany

**Keywords:** Amyloidosis, ATTR, Cardiomyopathy, Transthyretin, Treatment decisions

## Abstract

**Background:**

The efficacy of transthyretin stabilisation in cardiac transthyretin amyloidosis (ATTR-CM) has been demonstrated in a clinical trial setting, but little is known about treatment decision-making in the real world. Particularly, initiating or discontinuing specific therapy is challenging in early and advanced disease. We evaluated current decision pathways for tafamidis in ATTR-CM.

**Methods:**

This multicentre retrospective study included consecutive patients from 15 tertiary centres in Germany in whom ATTR-CM was newly diagnosed between January and June 2024, as well as patients, in whom tafamidis treatment was discontinued during this period.

**Results:**

Out of 516 patients with newly established ATTR-CM included in the present analysis, tafamidis was initiated in 414 (80%). The 99 patients without recommendation for tafamidis were older (*p* = 0.002), had a higher amyloidosis disease stage (NAC score), worse NYHA class (both *p* < 0.001), and higher NT-proBNP levels (*p* = 0.002) compared to those with tafamidis initiation. During the same observation period, tafamidis therapy was discontinued in 28 ATTR-CM patients. Treatment decisions were mainly taken by an interdisciplinary board (73% of centres). The most frequent reasons for not starting or stopping tafamidis were ‘frailty’ (47%/61%) and ‘life expectancy or comorbidity’ (38%/43%), respectively.

**Conclusions:**

In this multicentre analysis, treatment with tafamidis was initiated in about 80% of patients with newly diagnosed ATTR-CM. In most centres, treatment decisions were made by an interdisciplinary board, and the reasons for treatment decisions were similar across centres. Due to the lack of consensus criteria, our data may help to standardise decision pathways for ATTR-CM.

**Graphical Abstract:**

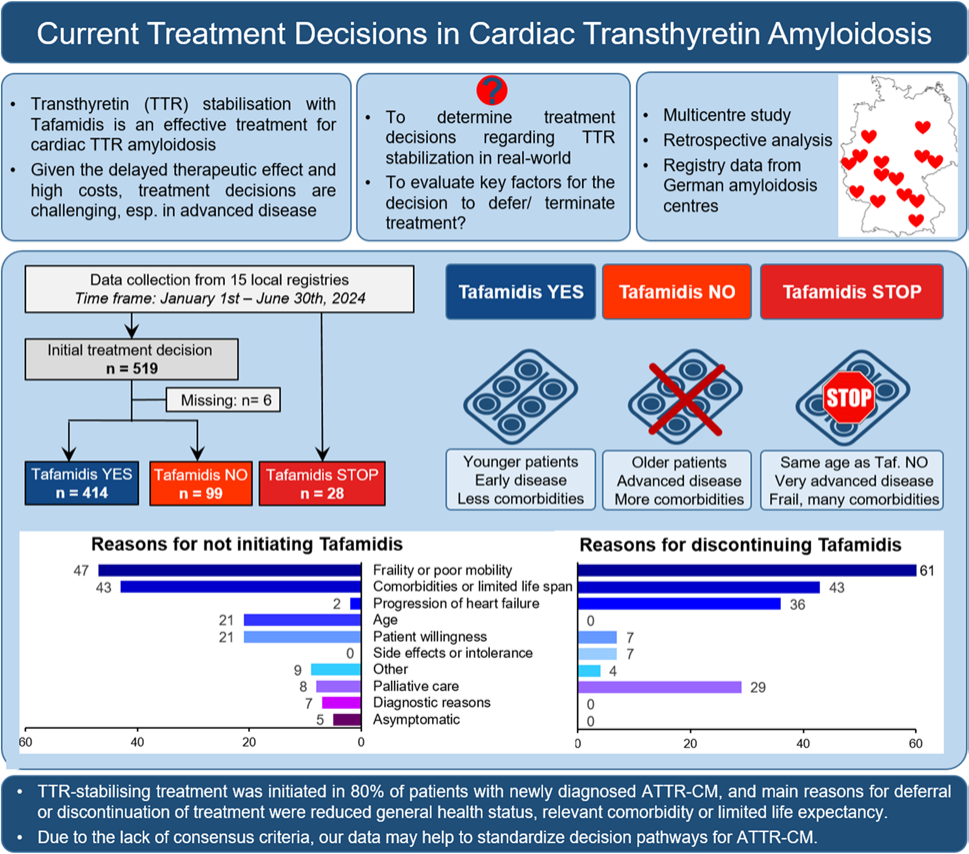

**Supplementary Information:**

The online version contains supplementary material available at 10.1007/s00392-026-02848-z.

## Introduction

Transthyretin amyloid cardiomyopathy (ATTR-CM) is an infiltrative disease characterised by the deposition of misfolded transthyretin (TTR) protein in the extracellular space. This leads to progressive myocardial dysfunction, conduction disorders, and to restrictive cardiomyopathy [1, 2]. Although historically considered rare, ATTR-CM is now recognised as a common cause of heart failure in elderly individuals, especially in cases presenting with left ventricular hypertrophy [3]. Advances in diagnostics, imaging modalities, and the availability of targeted therapies have led to an increased awareness of the disease.

The misfolding and aggregation of TTR protein can either occur due to amyloidogenic mutations in the *TTR* gene (ATTRv), or in the context of advanced age (wild-type or ATTRwt) [1]. In both ATTRv and ATTRwt, TTR dissociates from its (physiologic) tetrameric structure into monomers, that then aggregate to form amyloid fibrils [4]. Therapeutic approaches include stabilising the TTR protein in its tetrameric form (stabilisers), inhibiting TTR synthesis (gene silencers), and removing TTR depositions (depleters). Currently, TTR stabilisers and gene silencers have been proven efficacy in clinical trials, while studies regarding further gene silencers, gene therapy, and TTR with depleters are ongoing [5–8].

Prior to February 2025, the stabiliser tafamidis was the only medication specifically approved for the treatment of ATTRwt-CM. In the ATTR-ACT trial, tafamidis was associated with a reduced risk of all-cause mortality and cardiovascular hospitalizations as compared to placebo [5]. However, in patients with New York Heart Association (NYHA) class III, an indicator of advanced disease, rates of cardiovascular-related hospitalisations were increased, and the benefit of tafamidis was less pronounced compared to NYHA class I or II patients [5]. Retrospective analyses of real-world data have also confirmed the positive effects of tafamidis on worsening heart failure and mortality [9, 10].

Considering the patient characteristics in ATTR-ACT, the above-mentioned subgroup analyses and the delayed effect on survival, whereby the curves started to separate after 18 months of treatment [5] in a disease primarily affecting elderly individuals, patient selection seems crucial. This is particularly important given the high cost of tafamidis [11], the often major burden of comorbidities, and the recent approval of further targeted therapies, i.e. the stabiliser acoramidis and the silencer vutrisiran [6, 7].

The aims of this multicentre study were (1) to provide real-world data on prescription patterns of tafamidis in ATTR-CM patients at German tertiary care amyloidosis centres, particularly in cases of early and advanced disease, (2) to identify patient characteristics associated with treatment decisions to initiate, defer, or discontinue TTR-stabilising treatment, and (3) to evaluate treatment decision pathways and reasons for therapy initiation, possibly guiding treatment decisions in the future.

## Methods

This multicentre, retrospective, clinical registry study was performed across 15 tertiary referral centres in Germany. All sites obtained local ethical or institutional review board approval for their local registries. Diagnosis of ATTR-CM was confirmed according to the current ESC guidelines [2]. Consecutive patients were included if they presented to a cardiac amyloidosis centre due to confirmed transthyretin amyloidosis between January 1 st and June 30th 2024 (*cohort 1*: initial treatment decision cohort) or if an established specific treatment was discontinued during this period (*cohort 2*: treatment discontinuation cohort).

Data collection was based on standardised questionnaires assessing patients’ baseline characteristics, treatment decisions, and motivations for said decisions. Baseline characteristics included patient demographics, comorbidities, symptoms, heart rhythm, laboratory and echocardiographic values as well as diagnostic tests. All data were gathered from routine clinical care at each centre. Echocardiographic measures and bone scintigraphy results were determined according to current guideline recommendations. Variables not obtained during routine follow-up were considered as missing values.

A structured questionnaire with pre-defined answers assessing the local organisational structures and clinical reasoning for treatment decisions was completed by the physician representing the amyloidosis centre at each participating site (choice by menu item for individual patients, with option for multiple answers; for details, see supplemental material).

Collected and anonymized data from all centres was merged, checked for integrity (including signs for double-reporting), and analysed by the central study team. Statistical analysis was performed using Graph Pad Prism and Microsoft Excel. Categorical data are presented as absolute and relative frequencies and continuous data as median and interquartile range (IQR). Patients were categorised into three groups according to NYHA class (≤ I, II, ≥ III), NT-proBNP values (< 1000 ng/l, 1000–2999 ng/l, ≥ 3000 ng/l), and National Amyloidosis Centre (NAC) score (stage 1 = NT-proBNP ≤ 3000 ng/L and eGFR ≥ 45 ml/min; stage 3 = NT-proBNP > 3000 ng/L and eGFR < 45 ml/min; stage 2 = the remaining patients) [12]. Comparisons between groups were compared using the two-sided Mann–Whitney *U*-test for continuous variables or Fisher’s exact test for categorical variables. Two-sided *p*-values < 0.05 were considered as statistically significant.

## Results

### Initial treatment decision: cohort 1

**Fig. 1:**
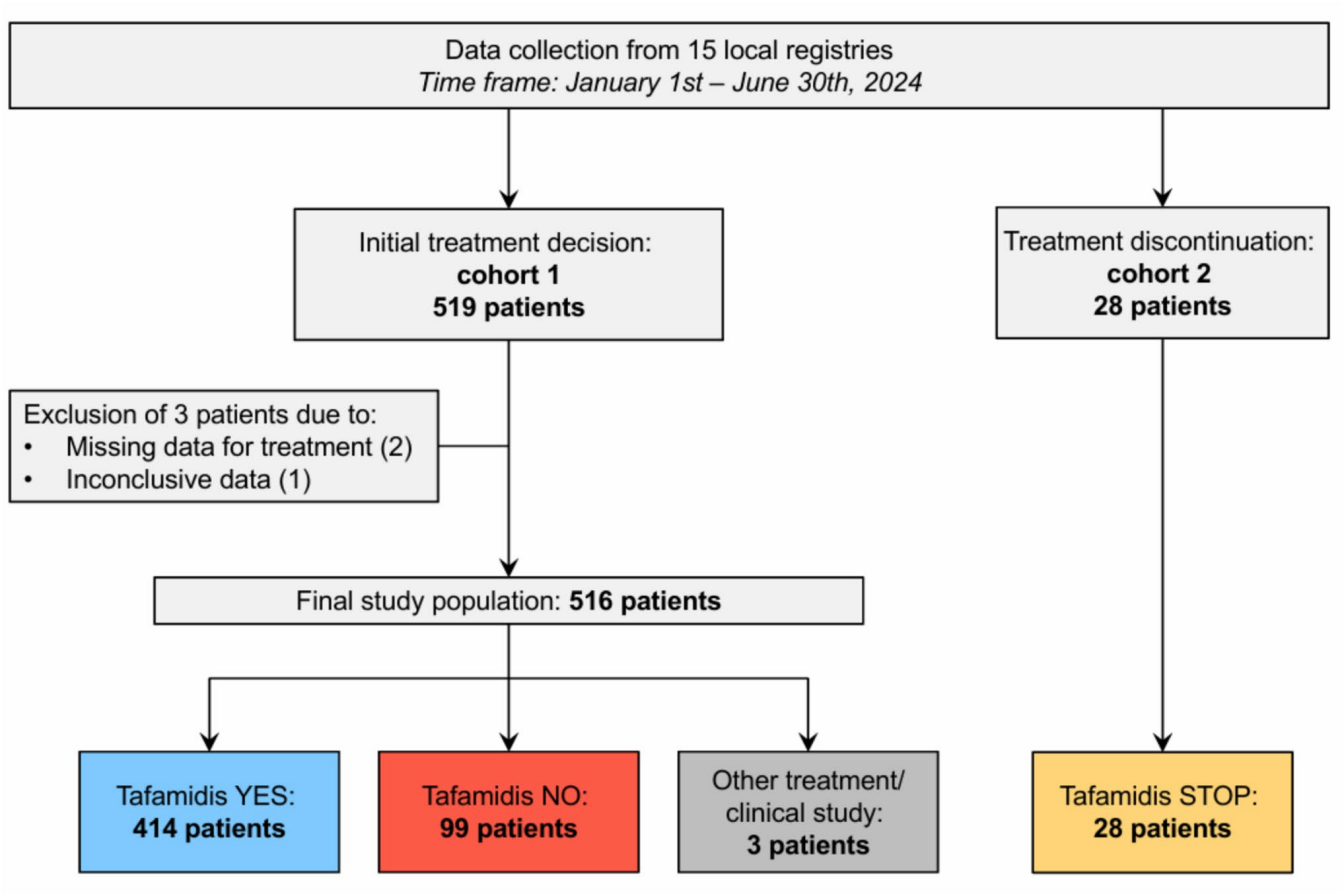
Study flow chart of cohort 1

Between January 1 st and June 30th 2024, 519 patients were recruited from 15 tertiary care amyloidosis centres. Of those, 3 patients were excluded due to missing or inconclusive data. Of the remaining 516 patients, 414 (80%) were initiated on specific treatment received (tafamidis YES), while 99 (19%) received no such recommendation (tafamidis NO), and 3 patients (0.6%) were treated with vutrisiran because of predominant neuropathy and ATTRv genotype (Fig. 1). Overall, the rates of tafamidis initiation varied between 44 and 96% between the participating centres (Table 1). There was no correlation between the overall volume of ATTR-CM patients and tafamidis prescription rates in each centre (*r*^2^ = 0.024, *p* = 0.58). The rates of treatment initiation were similar in wild-type, hereditary, and unknown genotype patients (80.6% vs. 78.6% vs. 81.6%, *p* = 0.91).

**Table 1:** Specific treatment initiation in cohort 1 according to centre

Centre no	*N*	Tafamidis YES	Tafamidis NO	Other treatment/clinical study	% Tafamidis YES
1	43	39	4	0	90.7
2	17	16	1	0	94.1
3	20	14	6	0	70.0
4	34	15	17	2	44.1
5	23	21	2	0	91.3
6	97	93	4	0	95.9
7	42	37	5	0	88.1
8	11	6	5	0	54.5
9	34	27	6	1	79.4
10	27	25	2	0	92.6
11	36	24	12	0	66.7
12	41	30	11	0	73.2
13	12	9	3	0	75.0
14	46	34	12	0	73.9
15	33	24	9	0	72.7
Total	**516**	**414**	**99**	**3**	**80.2**

Tafamidis YES, patients with newly established diagnosis of cardiac ATTR in whom tafamidis treatment was started; Tafamidis NO, patients with newly established diagnosis of cardiac ATTR in whom tafamidis therapy was not started

Baseline characteristics of the cohort 1 are shown in Table 2. The sample was representative of a typical ATTR-CM cohort, with a median age of 81 years (interquartile range, 76–84 years), a male predominance (88%), a high frequency of ATTRwt patients (3.3% had ATTRv). There was also a high rate of cardiovascular comorbidities. The median NT-proBNP level across the entire cohort was 2064 pg/ml (982–3934 pg/ml), > 80% were in NYHA class II or III, and echocardiography showed increased wall thickness, preserved ejection fraction and elevated left ventricular (LV) filling pressures. Five patients with ATTRv were diagnosed by a positive genetic test in the context of typical clinical and imaging findings of ATTR-CM, and one patient was diagnosed by tissue biopsy after carpal tunnel surgery.

**Table 2:** Characteristics of patients with newly established diagnosis of cardiac transthyretin amyloidosis (ATTR-CM)

	All patients	Tafamidis NO	Tafamidis YES	*p* value*
*N*	516	99	414	
Age, years	81 (76–84)	83 (78–85)	81 (75–84)	**0.0002**
Male, *n* (%)	451 (88)	84 (85)	367 (89)	0.392
Diagnostic modality, *n* (%)				**0.0242**
Bone scintigraphy	365 (70.7)	75 (75.8)	289 (69.8)	
Biopsy	145 (28.1)	21 (21.2)	123 (29.7)	
Other	6 (1.1)	3 (3.0)	2 (0.5)	
ATTR type, *n* (%)				0.909
ATTRwt	412 (79.8)	80 (80.8)	332 (80.2)	
ATTRv	17 (3.3)	3 (3.0)	11 (2.7)	
Unknown	87 (16.9)	16 (16.2)	71 (17.1)	
Heart failure severity				
NAC stage, *n* (%)				** < 0.0001**
1	290 (56.6)	37 (37.8)	251 (61.1)	
2	135 (26.4)	25 (25.5)	109 (26.5)	
3	87 (17.0)	36 (36.7)	51 (12.4)	
NYHA functional class, *n* (%)				** < 0.0001**
I	90 (17.5)	10 (10.3)	78 (18.9)	
II	284 (55.4)	35 (36.1)	248 (60.0)	
III	129 (25.1)	43 (44.3)	86 (20.8)	
IV	10 (1.9)	9 (9.3)	1 (0.2)	
NT-proBNP, pg/ml	2064 (982–3934)	3122 (1284–7527)	1974 (916–3550)	**0.0015**
eGFR, ml/min/1.73m^2^	60 (44–72)	44 (33–61)	60 (48–74)	** < 0.0001**
Comorbidities				
Atrial fibrillation, *n* (%)	253 (49.4)	55 (56.7)	197 (47.9)	0.142
Cancer, *n* (%)	81 (20)	23 (25.0)	58 (18.6)	0.185
Stroke, *n* (%)	51 (12.4)	14 (15.2)	37 (11.6)	0.371
CAD, *n* (%)	167 (40.5)	38 (40.4)	128 (40.3)	> 0.99
COPD, *n* (%)	34 (8.9)	9 (10.5)	25 (8.5)	0.527
Echocardiography				
LVEF,%	52 (47–57)	53 (45–55)	52 (48–58)	0.343
IVSD, mm	17 (15–20)	17 (14–19)	18 (16–20)	**0.0025**
E/e ‘	14 (10–18)	15 (11–20)	14 (10–18)	0.342

Categorical data are presented as absolute and relative frequencies, continuous data as median and interquartile range (IQR)

^*^Differences between tafamidis NO and tafamidis YES were tested with two-sided Mann–Whitney test for continuous variables or Fischer’s exact test for categorical variables

*Abbreviations*: *CAD*, coronary artery disease; *COPD*, chronic obstructive pulmonary disease; *eGFR*, estimated glomerular filtration rate; *ATTRv*, hereditary TTR amyloidosis. *IVSD*, inter-ventricular septal diameter; *LVEF*, left ventricular ejection fraction; *NAC*, National Amyloidosis Centre score; *NYHA*, New York Heart Association; *wtATTR*, wild-type TTR amyloidosis

Values in bold indicate significant differences

Patients in the tafamidis NO group were older (median 83 (78–85) vs. 81 (75–84) years, *p* = 0.001), and had more advanced disease with higher NT-proBNP levels (median 3122 (1284–7527) vs. 1974 (916–3550) pg/ml, *p* = 0.002), lower eGFR (median 44 (33–61) vs. 60 (48–74) ml/min/m^2^, *p* < 0.001), higher NAC scores (NAC stage 3 in 36% vs.12%, *p* < 0.001), and worse NYHA class (NYHA class III 44% vs 21%, *p* = 0.002) compared to tafamidis YES patients (Table 2). Patients receiving tafamidis were more likely to have received an invasive diagnosis by endomyocardial biopsy than other patients (21.2 vs. 29.7%, *p* = 0.024).

**Fig. 2:**
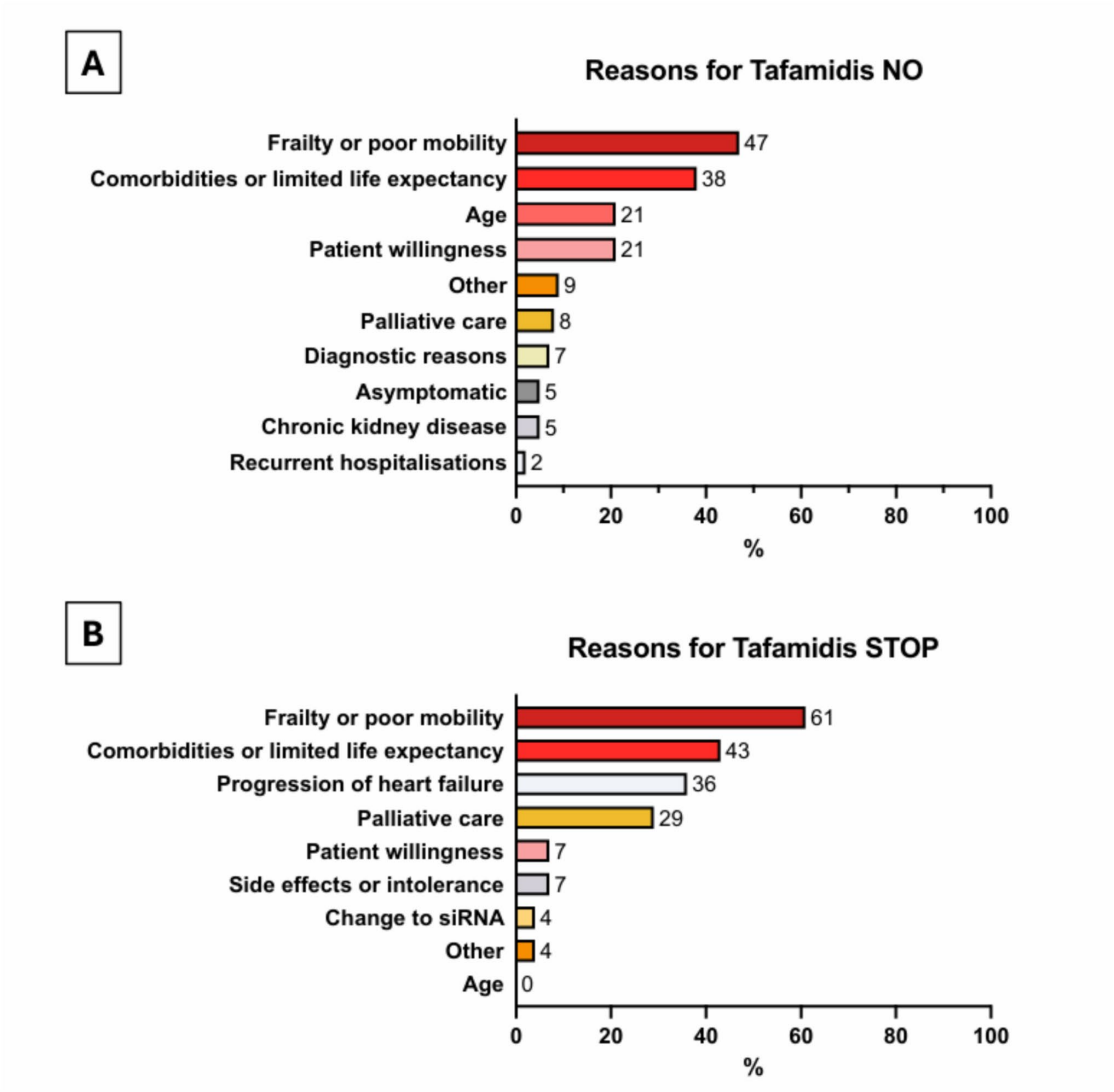
Treatment decisions. Reasons for individual treatment decisions for no initiation of tafamidis (tafamidis NO) (**A**) in cohort 1 or discontinuation of tafamidis (tafamidis STOP) (**B**) in cohort 2. Bar size indicates the percentage of patients in whom the respective reason was mentioned for individual treatment decision. Several items per patient could be chosen

The four most frequent reasons for not initiating tafamidis were ‘frailty or poor mobility’ (47%), ‘comorbidities or limited life expectancy’ (38%), ‘age’ (21%) and ‘patient willingness’ (21%) (Fig. 2A). Most decisions were based on more than one factor, i.e. the mean number of reasons for not initiating tafamidis was 1.7 per patient (except those who were asymptomatic). Only in four patients, all ≥ 89 years old, age was mentioned as sole reason for not initiating treatment. Frequent comorbidities that were recorded in the tafamidis NO group were neurological (25%), cardiac (20%), renal conditions (17%) and cancer (15%).

#### Tafamidis treatment in subgroups of patients with early and advanced disease

**Fig. 3:**
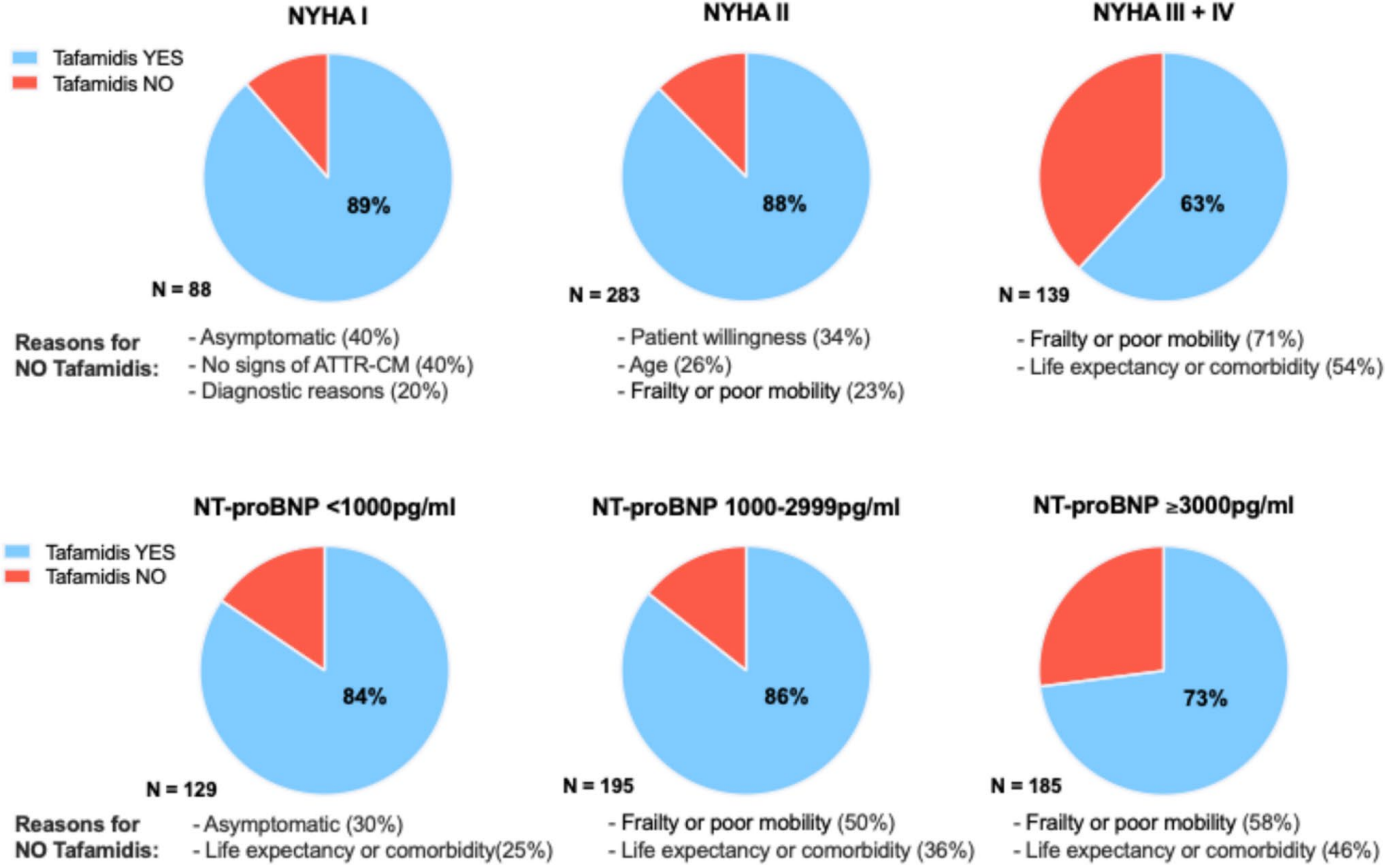
Tafamidis prescription patterns in cohort 1. Tafamidis prescription patterns according to New York Heart Association (NYHA) functional class and National Amyloidosis Centre (NAC) disease stage. ‘Diagnostic reasons’ indicate that a final diagnosis between AL and ATTR (or both) was not established

We analysed tafamidis initiation rates according to patient subgroups with early and advanced disease, respectively (Fig. 3). In patients with NYHA class I or NAC stage 1 (early disease stages), tafamidis initiation was recommended in 89% and 87%, respectively. As in the overall cohort, prescription rates varied between the centres, they were 40–100% in NYHA I and 33–100% in NAC 1 patients. In contrast, patients in NYHA III and IV or NAC stage 3 (advanced disease) received a recommendation for tafamidis therapy in 63% and 59% (both ranging from 0 to 100% between centres), respectively (Fig. 3). Similar results were observed in patients with NT-proBNP < 1000 pg/ml and ≥ 3000 pg/ml but with less pronounced differences regarding recommendations to initiate tafamidis (84% vs. 73%) (Supplement Figure S2).

The reasons against treatment with tafamidis were different in patients with early-stage disease (‘asymptomatic’ and ‘no signs of ATTR-CM’, both ≤ 40%), whereas ‘frailty or poor mobility’ and ‘comorbidities or limited life expectancy’ were the predominant reasons in patients with advanced disease (70% and 53%, respectively) (Fig. 3 and S2).

### Treatment discontinuation: cohort 2

Between January 1 st and June 30th 2024, discontinuation of tafamidis treatment (tafamidis STOP) was reported in 28 patients from 15 centres. The characteristics of patients in the tafamidis STOP group are compared to tafamidis NO (Table 3). Patients in the tafamidis STOP group were in a more advanced stage of disease, as indicated by higher NT-proBNP levels (median 6958 (3818–12,452) vs. 3122 (1284–7527) pg/ml, p = 0.002), lower LV ejection fraction (48 (40–54) vs. 53 (45–55) %, *p* = 0.042) and higher NAC stage (NAC stage 3 in 43% vs. 37%, *p* = 0.002). Furthermore, patients in the tafamidis STOP group had more comorbidities, such as atrial fibrillation (81% vs. 5%, *p* = 0.039), coronary artery disease (65% vs. 40%, *p* = 0.038) and chronic obstructive pulmonary disease (47% vs. 10%, *p* = 0.028). There was no significant difference in age or NYHA functional class between tafamidis STOP and tafamidis NO.

**Table 3:** Characteristics of tafamidis STOP vs. tafamidis NO

	Tafamidis STOP *N* = 28	Tafamidis NO *N* = 99	*p* value
Age, yrs	83 (80–85)	83 (78–85)	0.863
Male, *n* (%)	25 (89)	84 (85)	0.762
ATTR genotype, *n* (%)			0.907
ATTRwt	22 (78.6)	80 (80.8)	
ATTRv	1 (3.6)	3 (3.0)	
Unknown	5 (17.9)	16 (16.2)	
NAC stage, *n* (%)			**0.002**
1	2 (7.1)	37 (37.8)	
2	14 (50.0)	25 (25.5)	
3	12 (42.9)	36 (36.7)	
NYHA functional class, *n* (%)			0.202
I	0 (0)	10 (10.3)	
II	8 (28.6)	35 (36.1)	
III	16 (57.1)	43 (44.3)	
IV	4 (14.3)	9 (9.3)	
NT-proBNP, pg/ml	6958 (3818–12,452)	3122 (1284–7527)	**0.002**
eGFR, ml/min/1.73m^2^	40 (32–58)	44 (33–61)	0.442
Atrial fibrillation, *n* (%)	21 (80.8)	55 (56.7)	**0.039**
Cancer, *n* (%)	9 (42.9)	23 (25.0)	0.114
Stroke, *n* (%)	5 (22.7)	14 (15.2)	0.523
CAD, *n* (%)	15 (65.2)	38 (40.4)	**0.038**
COPD, *n* (%)	6 (47.4)	9 (10.5)	**0.028**
LVEF, %	48 (40–54)	53 (45–55)	**0.042**
IVSD, mm	19 (17–21)	17 (14–19)	**0.001**
E/e ‘	17 (13–22)	15 (11–20)	0.075

Categorical data are presented as absolute and relative frequencies, continuous data as median and interquartile range (IQR). Tafamidis STOP, patients in whom tafamidis therapy was discontinued; Tafamidis NO, patients with newly established diagnosis of cardiac ATTR in whom tafamidis therapy was not started. *Abbreviations*: *CAD*, coronary artery disease; *COPD*, chronic obstructive pulmonary disease; *eGFR*, estimated glomerular filtration rate; *ATTRv*, hereditary TTR amyloidosis. *IVSD*, inter-ventricular septal diameter; *LVEF*, left ventricular ejection fraction; *NAC*, National Amyloidosis Centre score; *NYHA*, New York Heart Association; *wtATTR*, wild-type TTR amyloidosis

Values in bold indicate significant differences

Supplement Table S1 shows the comparison between tafamidis YES and tafamidis STOP patients. Briefly, tafamidis STOP patients were older, suffered from more advanced disease in terms of NT-proBNP, kidney function, NAC score, LV remodelling, LV function, and comorbidities.

In tafamidis STOP patients, the most frequent reasons for tafamidis discontinuation (choice by menu item for individual patients) were ‘frailty or poor mobility’ (61%), ‘comorbidities or limited life expectancy’ (43%), ‘progression of heart failure’ (36%) or ‘palliative care’ (29%) (Fig. 2B). A change to siRNA treatment for amyloidosis was mentioned in 4%. Overall, age did not play a role for the discontinuation of tafamidis therapy.

### Structures of treatment decision

Seventy-three percent of centres reported that the initial decision to initiate tafamidis was made in a specific amyloidosis board. In 27% of centres, the treating physicians decided about specific ATTR treatment by themselves. Two centres with amyloidosis boards reported that the treating physician alone may initiate treatment decision about tafamidis in straightforward cases. Decisions about discontinuing treatment were also discussed in an amyloidosis board in 60% of centres. The treating physicians decided on discontinuation in 40% of centres. One centre reported treatment escalation or switching of therapies were discussed in the amyloidosis board, while the treating physician decided on discontinuation.

Out of a selection of clinical variables possibly influencing general treatment decisions, the most commonly mentioned in the questionnaire were NYHA class and frailty/mobility/distance in the 6-min-walking test (both reported by all centres) as factors influencing treatment initiation. Further important factors were the presence of relevant comorbidities (mentioned by 93% of centres), patient compliance to medication and follow-ups (93%), age (80%), NT-proBNP (60%), LV ejection fraction (60%), NAC stage (53%), and the number of hospitalisations related to heart failure (53%) (Supplemental Figure S1).

The reasons for treatment discontinuation most frequently mentioned were the presence of relevant comorbidities, frailty/mobility/distance in the 6-min-walking test (both 95%), NYHA class, and patient compliance (both 87%). Age (33%), ejection fraction (33%), and NAC stadium (20%) were considered in a small number of centres. The number of heart failure hospitalisations (73%) was important for deciding to discontinue treatment (Supplemental Figure S1).

## Discussion

This multicentre study assessed the prescription patterns of tafamidis for ATTR-CM in a real-world setting in Germany. The main findings are (1) prescription rates for tafamidis are currently about 80%; (2) amyloidosis centres exhibited high agreement in their reasoning for treatment decisions, despite different prescription rates; (3) patients, in whom initiation of tafamidis therapy was not recommended, were older and showed more advanced disease stages compared to those in whom treatment was initiated; (4) frailty and comorbidity burden were the most frequent reasons against tafamidis treatment at initial evaluation; (5) the absolute number of patients who discontinued tafamidis was low and was associated with signs of progressive disease or palliative care.

We provide a large contemporary analysis of real-world prescription patterns for tafamidis in ATTR-CM in Germany. Since the publication of the ATTR-ACT trial, few studies have analysed treatment decisions in a real-life setting. The characteristics of the patients in our analysis were similar to those in recent clinical trials involving a TTR stabiliser in ATTR-CM patients [6, 7].

### High prescription rates of TTR-stabilisation therapy in Germany

Small studies early after tafamidis approval revealed lower initiation rates of specific therapy. A Japanese study found that 46% of 83 patients were administered tafamidis between June 2019 and July 2021. The most frequent reasons for not prescribing tafamidis were advanced heart failure (ACC/AHA stage D or NYHA class III or IV) and frailty [13]. A single centre study including 107 ATTR patients between 2009 and 2021 found that 59% (*n* = 63) received tafamidis and that the main barriers to treatment were delays in obtaining the drug (31%) and monetary reasons (23%) [14]. In contrast, a single-centre study from Greece including 109 ATTR patients reported a prescription rate of 100% after tafamidis was approved in Greece [15]. An electronic healthcare records analysis found a prescription rate of 49% one year after approval of tafamidis 61 mg in the US [16], while another US multicentre health record study revealed that 40% ATTR-CM patients received tafamidis between January 2019 and May 2021 [9].

In Germany, two studies reported similar prescription rates (77% and 80%, respectively) to our study. Nies et al. showed that treatment was initiated in 77% of ATTR-CM patients between April 2020 and March 2021 [17]. In a single-centre observational study, tafamidis was initiated in 80% of patients between 2020 and 2022 [18]. Consistent with our findings of an 80% tafamidis prescription in 2024, the data show that overall initiation rates with tafamidis were similar over the last 4 years. In the meantime, evidence for specific therapy in ATTR-CM is growing [19] and diagnosis of ATTR-CM becomes earlier in the course of disease [20].

The higher prescription rates observed in our analysis may include a referral bias compared to analyses of electronic health care records, as patients with immobility or advanced disease stages might not be referred to tertiary amyloidosis centres for diagnostic workup and are less likely to be encouraged to undergo myocardial biopsy for confirmation of diagnosis. Furthermore, as the mortality reduction in the ATTR-ACT study occurred after approximately 18 months of therapy [5], some primary care physicians or ambulatory cardiologists may not refer patients who are elderly or have lifespan-limiting or multiple comorbidities. In other cases, patients themselves might refuse a complex and sometimes invasive diagnostic workup. This is supported by observations from our study and others [9, 10, 17].

### Reasoning for initial treatment decision in ATTR-CM

Despite the lack of general recommendations, the reasons for not initiating TTR-targeted treatment were similar across the centres. Overall, clinical status assessed by frailty, mobility and NYHA class was considered by all centres. Other important factors were the presence of—potentially—life-limiting comorbidities and age. This reflects an attempt to evaluate the overall health status and biological age. Patients’ preferences and compliance were also important factors. Age as the sole reason for not initiating TTR-stabilising treatment was given in only four patients, all ≥ 89 years old, an age with limited evidence for tafamidis treatment and high probability of futility [21]. Notably, severity scores in ATTR-CM such as the biomarker-based NAC score proposed by Gillmore et al. [12], was not generally used for treatment decisions. Dyspnoea was an important factor influencing treatment decisions, but this symptom may be affected by several cardiac or extracardiac comorbidities, such as atrial fibrillation, valvular heart disease or chronic obstructive pulmonary disease. Because of these limitations and the subjectiveness of NYHA class, a more thorough patient assessment, including a structured assessment of frailty using established scores such as the clinical frailty scale, is warranted for ATTR-CM patients, who are mostly multimorbid. Our data show that neither NYHA I and II are a clear indication for nor is NYHA III a strict contraindication against tafamidis. There was a mean of 1.7 reasons against tafamidis per patient suggesting an individualised treatment decision rather than standardised pathways.

A recent registry analysis showed that tafamidis treatment was associated with a lower NYHA class, while NT-proBNP and age revealed inconsistent results [17, 18]. The reasons given for not initiating tafamidis in the study from Nies et al. were advanced heart failure (24%), impaired general condition (15%), patient wish (11%), but 50% were due to unspecified reasons [17]. Another large amyloidosis registry did not report the reasons for the decision not to treat with tafamidis [18]. Thus, our analysis provides important clinical evidence regarding the current reasoning for and against specific therapies for amyloidosis. These data may help to develop standardised criteria for treatment decisions in ATTR-CM, which is currently lacking in guidelines recommendation [22].

In fact, extensive treatment evaluation is performed due to the high cost of tafamidis, which motivates physicians trying to select patients who may benefit from treatment. This approach of precision medicine requires significant human and structural resources at each centre. Furthermore, it is different to most therapeutic concepts in cardiovascular medicine, where drugs were applied to all eligible patients, for instance heart failure with reduced ejection fraction.

### Treatment discontinuation in ATTR-CM

Few studies have evaluated termination of treatment. A single-centre German study reported that therapy was discontinued in 5 patients after thorough interdisciplinary discussion. The reasons for treatment discontinuation were new malignancy (2 patients), end-stage renal insufficiency (1 patient), and terminal heart failure (2 patients) [18].

The decision to terminate a specific treatment may be particularly difficult, due to its potential psychological impact on patients, their families, and the treating physicians [23]. Although both, not initiating and discontinuing treatment, effectively mean transitioning into a palliative care setting, termination is perceived as more drastic. In our analysis, termination of tafamidis was rare and was associated with older age and advanced heart failure, particularly compared to those patients who did not receive tafamidis at the initial evaluation. Thus, the threshold for discontinuing treatment appears to be even higher than for not starting treatment. The reasons for treatment discontinuation indicated by the centres were similar to those that influenced the decision to initiate treatment. An exception was age (mentioned by 33% of centres), which probably reflects the greater importance of actual health status over numerical age. In contrast, recurrent heart failure hospitalisations, i.e. progression of ATTR-CM supposing futility of further treatment, were a frequent reason for discontinuing tafamidis.

Together with the limited evidence from other studies, the most important individual reasons for tafamidis discontinuation were progressive heart failure and a change to palliative care. These results may help to inform future treatment decisions.

### Clinical perspective

The steep increase in ATTR-CM diagnoses at all stages of the disease and the lack of consensus criteria for treatment decision pose challenges in daily clinical practice. Current ESC guidelines only provide NYHA functional class only for patient selection [24]. Conversely, evidence for the beneficial effects of tafamidis in advanced stages is growing [25, 26]. Tertiary care centres have developed local expert panels to discuss treatment initiation, particularly in grey areas, such as NYHA class III or advanced age. Alongside these local institutions, which make individual treatment decisions, standardised pathways and criteria for patient selection are needed to support decision-making, particularly with regard to the initiation and discontinuation of specific therapies [19]. Our data could represent a significant step forward in the global puzzle for advancing precision medicine in ATTR-CM. Considering the recent approval of new therapeutic options, our data provide helpful insights in the decision-making process, which will also apply to acoramidis and vutrisian aiming to stop disease progression. Since the number of patients is growing, decision-making will remain the key issue.

### Limitations

This was a retrospective registry study, which has inherent limitations. However, the inclusion of consecutive patients over 6 months from 15 centres may provide robust evidence. The data might be affected by reporting bias, especially the underreporting of patients who did not receive or discontinued TTR-targeted treatment outside the participating centres. Patients diagnosed in primary care centres may not be referred or may be treated differently for a variety of patient specific factors leading to the wide range in disease stages, comorbidity burden and, hence, prescription rates. As such, patients with very advanced heart failure or geriatric patients might be underdiagnosed and will therefore not be represented in this study. These limitations particularly apply for patients with inconclusive results of non-invasive testing, in whom cardiac biopsy was not pursued for any reason. Different approaches to the final diagnosis may apply for different prescription rates among centres. For example, patients with inconclusive non-invasive imaging findings may not always undergo invasive biopsy, particularly if they are elderly or ‘frail’. This is supported by the higher rate of biopsy-proven diagnosis in the tafamidis YES group compared to tafamidis NO group. Another limitation is the low number of patients with ATTRv included in this study (without genetic specification), often due to pending results of the genetic testing. Our study focused on TTR-stabilising therapy and did not assess concomitant heart failure medication. The time to diagnosis, duration of prior tafamidis treatment was well as the exact rates of tafamidis discontinuation were not available.

## Conclusions

This multicentre, registry-based analysis in Germany found that 80% of patients with ATTR-CM were prescribed tafamidis in 2024. Treatment decisions are consistent between centres and are mainly based on board structures. The most frequent reasons for not prescribing tafamidis were comorbidities and frailty. The findings of this study could help to standardise treatment decision pathways for the initiation and discontinuation of specific amyloidosis therapy in a growing patient population diagnosed with ATTR-CM.

## Supplementary Information

Below is the link to the electronic supplementary material.

ESM 1 (PDF 600 KB)

## Abbreviations

ATTR-CM: Transthyretin amyloid cardiomyopathy
ATTRv: Variant or hereditary ATTR
ATTRwt: Wild-type ATTR
CAD: Coronary artery disease
COPD: Chronic obstructive pulmonary disease
eGFR: Estimated glomerular filtration rate
IVSD: Inter-ventricular septal diameter
LVEF: Left ventricular ejection fraction
NAC: National Amyloidosis Centre
NT-proBNP: N-terminal -pro hormone brain natriuretic peptide
NYHA: New York Heart Association
TTR: Transthyretin
siRNA: Small interfering ribonucleic acid
