# Supplementary material for: Current treatment decisions in cardiac transthyretin amyloidosis: a multicentre analysis
Source: Clin Res Cardiol. 2026 Jan 26;115(5):862–74. doi: 10.1007/s00392-026-02848-z (PMC13083537; doi:10.1007/s00392-026-02848-z)
Supplement: Supplementary file 1 — (PDF 600 KB) [file 392_2026_2848_MOESM1_ESM.pdf]

SUPPLEMENTARY INFORMATION

Table S1: Characteristics of Tafamidis STOP vs. Tafamidis YES

	Tafamidis STOP N=28	Tafamidis YES N=414	p value
**Age, yrs**	83 (80-85)	81 (75-84)	**0.012**
**Male, n (%)**	25 (89)	367 (89)	>0.99
**ATTR genotype, n (%)**			0.748
**ATTRwt**	22 (78.6)	332 (80.2)	
**ATTRv**	1 (3.6)	11 (2.7)	
**Unknown**	5 (17.9)	71 (17.1)	
**NAC stage, n (%)**			**<0.0001**
**1**	2 (7.1)	251 (61.1)	
**2**	14 (50.0)	109 (26.5)	
**3**	12 (42.9)	51 (12.4)	
**NYHA functional class, n (%)**			**<0.0001**
**I**	0 (0)	78 (18.9)	
**II**	8 (28.6)	248 (60.0)	
**III**	16 (57.1)	86 (20.8)	
**IV**	4 (14.3)	1 (0.2)	
**NT-proBNP, pg/ml**	6958 (3818-12452)	1974 (916-3550)	**<0.0001**
**eGFR, ml/min/1.73m^2**	40 (32-58)	60 (48-74)	**<0.0001**
**Atrial fibrillation, n (%)**	21 (80.8)	197 (47.9)	**0.014**
**Cancer, n (%)**	9 (42.9)	58 (18.6)	**0.020**
**Stroke, n (%)**	5 (22.7)	37 (11.6)	0.169
**CAD, n (%)**	15 (65.2)	128 (40.3)	**0.027**
**COPD, n (%)**	6 (47.4)	25 (8.5)	**0.006**
**LVEF, %**	48 (40-54)	52 (48-58)	**0.006**
**IVSD, mm**	19 (17-21)	18 (16-20)	**0.043**
**E/e‘**	17 (13-22)	14 (10-18)	**0.008**

Table S1: Characteristics of Tafamidis STOP vs. Tafamidis YES

Tafamidis STOP, patients in whom Tafamidis therapy was discontinued; Tafamidis Yes, patients in whom Tafamidis was initiated after diagnosis of ATTR.

Categorical data are presented as absolute and relative frequencies, continuous data as median and interquartile range (IQR). Abbreviations: CAD, coronary artery disease; COPD, chronic obstructive pulmonary disease; eGFR, estimated glomerular filtration rate; ATTRv, hereditary TTR amyloidosis. IVSD, inter-ventricular septal diameter; LVEF, left ventricular ejection fraction; NAC, national amyloidosis center score; NYHA, New York Heart Association; wtATTR, wild-type TTR amyloidosis.

Figure S1: Factors affecting for treatment decisions

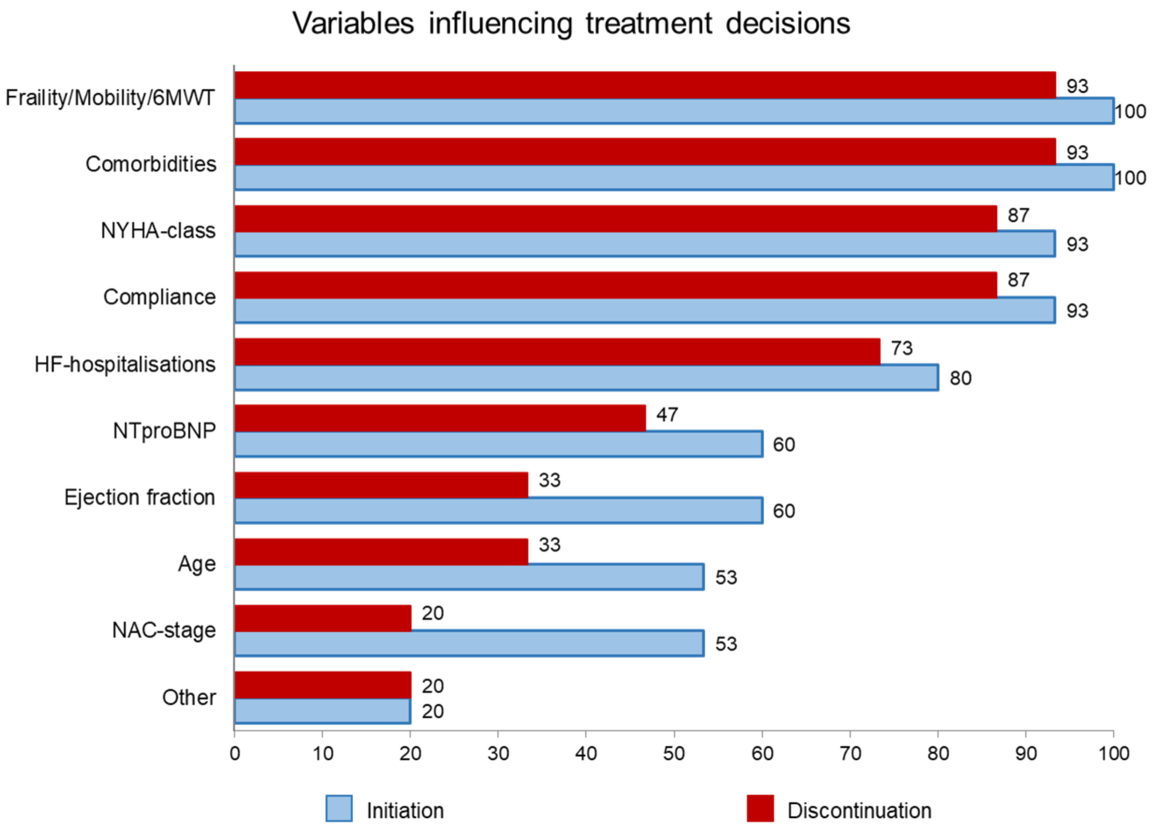

Figure S1: Factors affecting treatment decisions

Reasons for treatment decisions for initiation in cohort 1 (red) or discontinuation in cohort 2 (blue) of tafamidis therapy according to questionnaires response. Bar size indicates the percentages of centres that mentioned the respective variable as relevant for their treatment decision.

Figure S2: Tafamidis prescription patterns according to NT-proBNP values

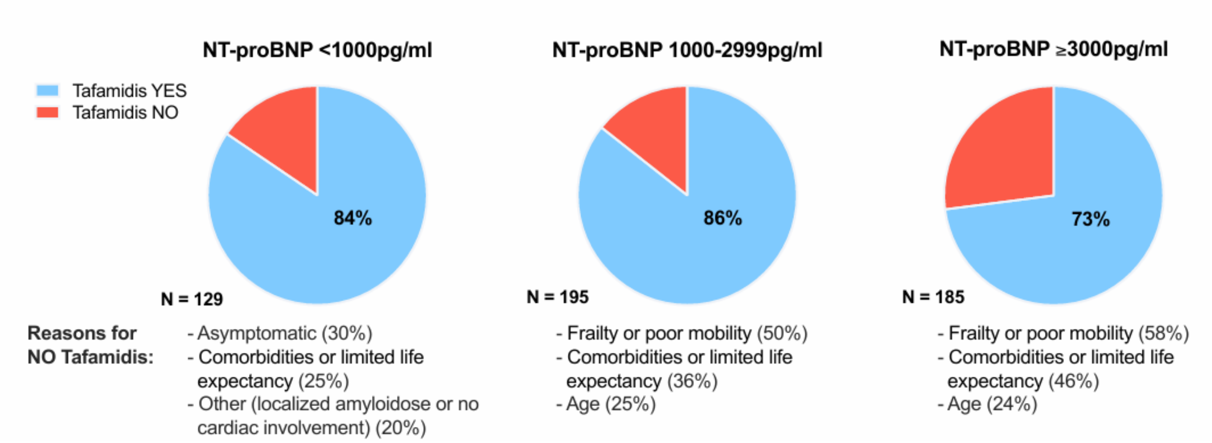

Figure S2: Tafamidis prescription patterns according to NT-proBNP values

Tafamidis prescription patterns according to NT-proBNP values.

Supplementary Information: Questionnaires

Variables of the standardised questionnaires assessing patients' baseline characteristics, treatment decisions and motivations for said decisions:

- Amyloidosis Center
- Age (years)
- Sex (m / f)
- NYHA class
- NT-proBNP (ng/L)
- eGFR (ml / min)
- Ejection fraction (%)
- IVSD (mm)
- E/E'
- Atrial Fibrillation (y / n)
- Cancer (y / n)
- Stroke (y / n)
- CAD (y / n)
- COPD (y / n)
- Other disabling or life limiting comorbidities (multiple answers allowed)
- Diagnosis (scintigraphy =1; biopsy =2; other =3)
- ATTR-CM (1=wtATTR; 2=hATTR)
- Specific treatment (0 = none; 1 = tafamidis; 2 = other or in clinical study)
- Only for treatment-initiation:
    - Reason for no tafamidis (1=age; 2=poor mobility / frailty; 3=life expectancy or relevant comorbidity; 4=patient willingness; 5= palliative situation; 6=other)
- Only for treatment-discontinuation:
    - Specific treatment since when? (date)
    - Specific treatment stopped when? (date)
    - Reason for stop of tafamidis / specific treatment (1=age; 2=poor mobility / frailty; 3=life expectancy or relevant comorbidity; 4=patient willingness; 5= palliative Situation; 6=recurrent hospitalisations; 7=relevant side effects / intolerance; 8 =other)

Variables of the structured questionnaire assessing the local organisational structures and clinical reasoning for treatment decisions:

- The initial treatment decision in our centre is based on
    - the decision of the treating physician
    - the decision of an Amyloidosis Board
    - other
- The follow-up treatment decision (continuation / discontinuation of specific treatment, switch of therapy) is based on:

    - the decision of the treating physician
    - the decision of an Amyloidosis Board
    - other
- Factors relevant for initial decision (multiple selection possible)
    - Age
    - NYHA class
    - NT-proBNP
    - Ejection fraction
    - Gilmore Stage
    - Frailty / Mobility / Distance in 6-minute-walk-test
    - Life-limiting Comorbidities
    - Number of hospitalisations for HF
    - Patient compliance
    - Other
- Factors relevant for continuation / switch / discontinuation of therapy (multiple selection possible)
    - Age
    - NYHA class
    - NT-proBNP
    - Ejection fraction
    - Gilmore Stage
    - Frailty / Mobility / Distance in 6-minute-walk-test
    - Life-limiting Comorbidities
    - Number of hospitalisations for HF
    - Patient compliance
    - Other
